# Impact of whole-grain interventions on serum vitamin D levels in individuals at risk of diabetes

**DOI:** 10.3389/fnut.2025.1658961

**Published:** 2025-10-23

**Authors:** TingTing Liu, LiMing Wang, LingLing Ou, Jing Feng, JiaLi Cheng, ZhaoLong Gong

**Affiliations:** ^1^National Institute for Nutrition and Health, Chinese Centre for Disease Control and Prevention, Beijing, China; ^2^Hospital of Beijing Institute of Technology, Beijing, China

**Keywords:** whole grains, 25-hydroxyvitamin D, intervention, prediabetic population, vitamin D status

## Abstract

**Introduction:**

We examined the effects of a whole-grain intervention on serum vitamin D levels in pre-diabetic individuals in China.

**Methods:**

Participants aged 18–65 years at risk of diabetes participated in a 12-week randomised parallel controlled trial. Intervention groups consumed 50 g/day and 100 g/day of whole grains, whereas the control group maintained a regular diet. All participants received nutritional guidance. Serum 25-hydroxyvitamin D levels were monitored at baseline, 6 weeks, and 12 weeks using liquid chromatography–tandem mass spectrometry.

**Results:**

At 6 weeks, serum 25-hydroxyvitamin D levels remained insufficient across all groups. By 12 weeks, both intervention groups showed significant increases, reaching adequate levels, whereas the control group remained at the lower limit of adequacy.

**Conclusion:**

Sustained whole-grain intake improves vitamin D status in pre-diabetic individuals.

## 1 Introduction

Diabetes mellitus is one of the most pressing public health concerns worldwide. It currently affects approximately 529 million adults, with projections suggesting that this number will continue to rise to 1.31 billion by 2050. In China, over 118 million individuals are living with diabetes. Since the pre-diabetic stage is often asymptomatic, many individuals remain undiagnosed (1, 2). Compared to individuals with normal blood glucose levels, the life expectancy of patients aged 40 is reduced by an average of 4.2 years in those with diabetes and 0.7 years in those with prediabetes (3). Dietary modification is required for the prevention and management of diabetes. In this context, whole-grain diets, characterised by high dietary fibre, low fat, and low calorie content, have shown particular promise (4). Unlike refined grains, whole grains contain the bran, germ, and endosperm of the original grain, making them rich in nutrients and bioactive ingredients such as dietary fibre, B vitamins, polyphenols, and minerals. Evidence from prospective studies suggests that the bioactive properties of whole grains are associated with a reduced risk of several metabolic diseases (including diabetes), with possible mechanisms of action comprising anti-inflammation, antioxidation, and cryoprotection (5, 6).

In the nutritional metabolism process of the organism, vitamin D—a fat-soluble vitamin—is essential to maintain bone health and immune system function. However, approximately 50.0% of the global population suffers from vitamin D deficiency owing to several factors, including insufficient sunlight exposure and inadequate dietary intake. This is especially common at higher latitudes (Northern Europe and East Asia), where the duration of exposure to sunlight is shorter and, therefore, vitamin D intake is lower (7). Adequate vitamin D levels help prevent and manage metabolic syndromes, including diabetes mellitus, as vitamin D exhibits indirect antioxidative properties and helps maintain normal resting ROS levels. Vitamin D reduces inflammation, regulates Ca^2+^ homeostasis, and reduces insulin resistance, which improves glucose and lipid metabolism in insulin-sensitive tissues (8, 9).

Emerging evidence highlights the role of whole grains in promoting metabolic health. However, the interplay between whole-grain dietary patterns and vitamin D homeostasis remains unexplored. To date, no study has specifically explained how sustained whole-grain intake modulates serum vitamin D levels, particularly in patients with diabetes, a demographic with heightened risks of micronutrient insufficiency. This randomised controlled trial bridges this critical knowledge gap by systematically evaluating the effect of a whole grain-enriched diet on the vitamin D status amongst adults in the pre-diabetic stage. Our findings showed that community-acceptable dietary modifications involving whole grains significantly elevated circulating vitamin D concentrations. These results provide empirical support for integrating whole-grain interventions into government-led nutrition programmes and offer practical foundations for clinical guidelines and public health policies targeting the dual burdens of diabetes and vitamin D deficiency.

## 2 Materials and methods

### 2.1 Study design and population

This randomised parallel-controlled intervention trial was conducted at the Beijing Institute of Technology University Hospital between June and September in Beijing. Participants were recruited from the outpatient clinics of community hospitals. Eligible participants were aged 18–65 years and met at least one of the diagnostic criteria for pre-diabetes as defined by the ‘Standards of Medical Care in Diabetes-2023’: fasting blood glucose 5.6–6.9 mmol/L or glycated haemoglobin (HbA1c) 5.7–6.4%. All participants completed a signed informed consent form prior to enrolment. Participants were excluded if they had gastrointestinal disorders; tumours; diabetes mellitus; cardiovascular or cerebrovascular diseases; cardiac, hepatic, or renal insufficiency; severe infectious diseases; autoimmune diseases; used antihypertensive, hypoglycaemic, or hypolipidaemic drugs; were pregnant or preparing for pregnancy; took supplements containing multivitamins, fish oils, n-3 fatty acids, or other nutrients continuously for 1 month; were allergic to whole grains or consumed more than 50 g of whole grains daily; or were unable to complete the study, as determined by the investigator.

Using PASS 15 software, the required sample size was calculated as 30 participants per group (1:1:1), based on a bilateral alpha level of 0.05 and a test power of 90.0%. Allowing for a 20.0% dropout rate, the target sample size was increased to 38 participants per group. A total of 147 individuals at risk for prediabetes were recruited through on-site screening of fasting blood glucose and glycated haemoglobin, considering factors such as their willingness to participate and comply. Participants were randomly divided into three groups: intervention group A (*n* = 48), intervention group B (*n* = 50), and control group C (*n* = 49).

#### 2.1.1 Intervention methods

Participants in intervention groups A and B consumed 50 g/day and 100 g/day of whole grains, respectively, whereas those in control group C maintained their usual dietary habits. Low glycaemic index whole-grain foods were provided in the form of whole wheat steamed buns and pre-cooked brown rice, produced and standardised by the Hebei Tongfu Group. To ensure quality and compliance, food items were distributed to participants every 2 weeks. The specific ingredients of this formula are listed in Table 1. Participants received whole grains according to their dietary preferences. In accordance with the requirements of the dosage group, participants selected and consumed their food every day, with reference to the whole grain content of each food, and recorded their consumption.

**Table 1:** Specific formula ingredients of whole grains.

Serial number	Whole-grain food	Specific formula ingredients	Content of whole grains
1	50% whole-grain steamed buns	Wheat flour and whole wheat flour	25 g per bun
2	100% whole-grain steamed buns	Whole wheat flour	50 g per bun
3	Quinoa and brown rice	Brown rice, oats, and red quinoa	50 g per bowl
4	Red rice and brown rice	Brown rice, oats, and red rice	50 g per bowl

This study was reviewed and approved by the Ethics Committee of the Institute of Nutrition and Health, Chinese Centre for Disease Control and Prevention (Approval No. 2023–002). All participants provided written informed consent.

### 2.2 Sample collection and processing

#### 2.2.1 Collection and processing of blood samples

After an overnight fast for 8–12 h, 3–5 mL of venous blood was drawn from the antecubital vein of the participants in a seated position during the early morning. All blood collections were performed by professional physicians on-site, who also managed any special situations. Blood samples were kept at room temperature for 20–30 min and were then centrifuged at 3,000 rpm for 10 min. The serum was separated and pipetted into the corresponding cryopreservation tubes in the absence of light. The serum samples were placed in a −80 °C refrigerator for storage.

#### 2.2.2 Reagents and instruments

The reagents used for the study include 25-hydroxyvitamin D_3_ (25-OH VD_3_); 25-hydroxyvitamin D_2_ (25-OH VD_2_); 25-OH VD_3_ (6,19,19-d3); and 25-OH VD_2_ (6,19,19-d3) (Lot Nos. 17938, 17937, 705888, 705497, Sigma, United States). The quality control items used in this study include multiple Quality Control samples (QCs) for fat-soluble vitamins (Batch No. 20230304, Beijing HuadaJibi Ai Biotechnology Co.); formic acid (Batch No. A117-50, Fisher Scientific, Germany), hexane (Batch No. 1.04372.0500, Merck, Germany), methanol (Batch No. 1.06007.4008, Merck, Germany), and amine formate (Batch No. 540–69-2, Merck, Germany). The pure water used in the experiment was obtained from a Milli-Q water purifier.

The instruments used were a liquid chromatography-triple quadrupole mass spectrometer (model: Shimadzu 8,060, Shimadzu Corporation, Japan) and a high-speed centrifuge (Beckman Corporation, United States).

#### 2.2.3 Measurement of serum samples

##### 2.2.3.1 Pretreatment

Serum sample of 100 μL was mixed well with 10 μL of the internal standard working preparation solution (with a concentration of 200 ng/mL) and 300 μL of methanol. The mixture was shaken at 1,500 rpm for 30 min and then allowed to stand at room temperature. Subsequently, 750 μL of n-hexane was added to the solution. It was shaken at 1,500 rpm for 30 min and centrifuged at 12,000 rpm for 10 min. After the completion of the process, the sample was divided into three layers. The top organic layer was placed in another centrifuge tube (caution was taken to ensure that the middle layer was not touched), and nitrogen was blown on it at room temperature. Afterwards, 100 μL of the compound solution was added to the tube and mixed thoroughly at 1,500 rpm for 3 min. It was then taken out and transferred to a lined tube for instrumental measurement (10). Pre-treatment experiments for the standard curve and QC samples were performed in the same manner.

##### 2.2.3.2 Chromatography and mass spectrometry (MS) conditions

The chromatography conditions were as follows: chromatographic column: C18 ACQUITY UPLC BEH C18 1.7 um 2.1 × 50 mm; column temperature: 30 °C; flow rates: 0.4 mL/min; mobile phase: A-methanol (containing 0.1% formic acid and 5 mmol/L ammonium formate) and B-water (containing 0.1% formic acid and 5 mmol/L ammonium formate); elution conditions: 0 min set to 20% B-phase, 0.50 to 2.50 min set to 10% B-phase, 2.51 to 7.50 min change to 0% B-phase, and 7.51 to 10.00 min change to 20% B-phase; and injection volume: 2 μL. The mass spectrometry conditions were as follows: the ion source was electrospray ionisation (ESI), positive ion mode; desolventization temperature: 500 °C; heating gas flow: 10 L/min; nebulizing gas flow rate: 3 L/min; heating block temperature: 400 °C; and drying gas flow rate: 10 L/min. The parent and daughter ions with the largest responses were selected as quantitative ion pairs. The specific mass spectrometer instrument parameters are listed in Table 2.

**Table 2:** Setting parameters of the mass spectrometer instrument.

Serum to be measured	Precursor parent ion (m/z)	Quantum ion (m/z)	Q1 predeviation (V)	Crash voltage (V)	Q3 predeviation (V)
25-OH Vitamin D3	401.1	365.3	−11.0	−12.0	−25.0
25-OH Vitamin D3-d3	404.1	386.3	−11.0	−12.0	−18.0
25-OH Vitamin D2	413.1	355.3	−18.0	−14.0	−24.0
25-OH Vitamin D2-d3	416.2	398.2	−18.0	−13.0	−29.0

### 2.3 Data processing and analysis

Data were expressed as mean ± standard deviation or quartiles, as appropriate. A one-way analysis of variance and rank sum tests were used to analyse the differences between groups and within groups at different periods. Data were processed and analysed using SAS (v.9.4) and R 4.4.1, and the differences were considered statistically significant at a *p*-value of <0.05.

## 3 Results

### 3.1 General information

All participants demonstrated high adherence to the study protocol throughout the intervention period. Based on dietary records, participants in the intervention groups consistently consumed whole grain foods according to their assigned dosage (50 g/day for group A and 100 g/day for group B), whereas those in the control group C maintained their regular diet.

Additionally, 25-OH VD is the main circulating form of vitamin D in the blood and a reliable indicator of the nutritional status of vitamin D in the body. It consists of 25-OH VD_2_ and 25-OH VD_3_, which are the main circulatory forms of vitamin D in the blood.

According to the Health Industry Standard of the People’s Republic of China—Screening Methods for Vitamin D Deficiency in the Population (WS/T 677–2020), serum 25-OH VD levels ≥20 ng/mL indicate adequacy, 12 ~ 20 ng/mL indicate insufficiency, and <12 ng/mL indicate deficiency.

The Kolmogorov–Smirnov normality test indicated that 25-OH VD_3_ and 25-OH VD concentrations in each study group conformed to a normal distribution (*p* > 0.05), and the normality of 25-OH VD_2_ in serum was a *p*-value of < 0.05; therefore, mean ± standard deviation (x̄ ± s) and quartiles were used to express them, respectively. 25-OH VD_2_ levels remained stable and low across all groups. Given that 25-OH VD_3_ constitutes the majority of circulating vitamin D, total 25-OH VD trends closely mirrored those of 25-OH VD_3_ (Table 3).

**Table 3:** Baseline, interim, and final vitamin D levels (ng/mL).

Group*	25-OH VD3	25-OH VD2	25-OH VD	25-OH VD
	x̄ ± s	M(Q1,Q3)	x̄ ± s	M(Q1,Q3)
A1 (*N* = 48)	15.24 ± 4.06	1.14 (0.80,1.56)	16.20 ± 4.25	16.24 (13.20,19.33)
A2 (*N* = 48)	15.57 ± 5.19	1.25 (0.95,1.57)	16.40 ± 5.10	15.40 (12.82,20.96)
A3 (*N* = 47)	20.86 ± 6.21	1.14 (0.82,1.57)	21.92 ± 6.44	21.98 (17.49,25.87)
B1 (*N* = 50)	15.11 ± 5.46	1.13 (0.81,1.66)	15.83 ± 5.90	14.50 (11.06,19.16)
B2 (*N* = 46)	14.10 ± 5.08	1.11 (0.89,1.77)	14.99 ± 5.24	13.34 (10.80,20.32)
B3 (*N* = 45)	22.72 ± 8.30	1.12 (1.00,1.65)	24.04 ± 8.39	21.93 (18.22,28.27)
C1 (*N* = 49)	15.42 ± 6.34	1.14 (0.86,2.07)	16.20 ± 6.24	14.03 (11.32,22.03)
C2 (*N* = 45)	13.44 ± 5.42	1.09 (0.81,1.56)	14.35 ± 5.38	13.98 (10.02,17.52)
C3 (*N* = 47)	19.42 ± 6.48	1.44 (1.11,2.14)	20.52 ± 6.40	19.71 (15.34,25.80)

* *N* denotes the number of samples retained in each group at each intervention stage. A, B, and C denote intervention groups A, B, and C, respectively. 1, 2, and 3 represent baseline, 6 weeks of intervention, and 12 weeks of intervention, respectively.

**Figure 1:**
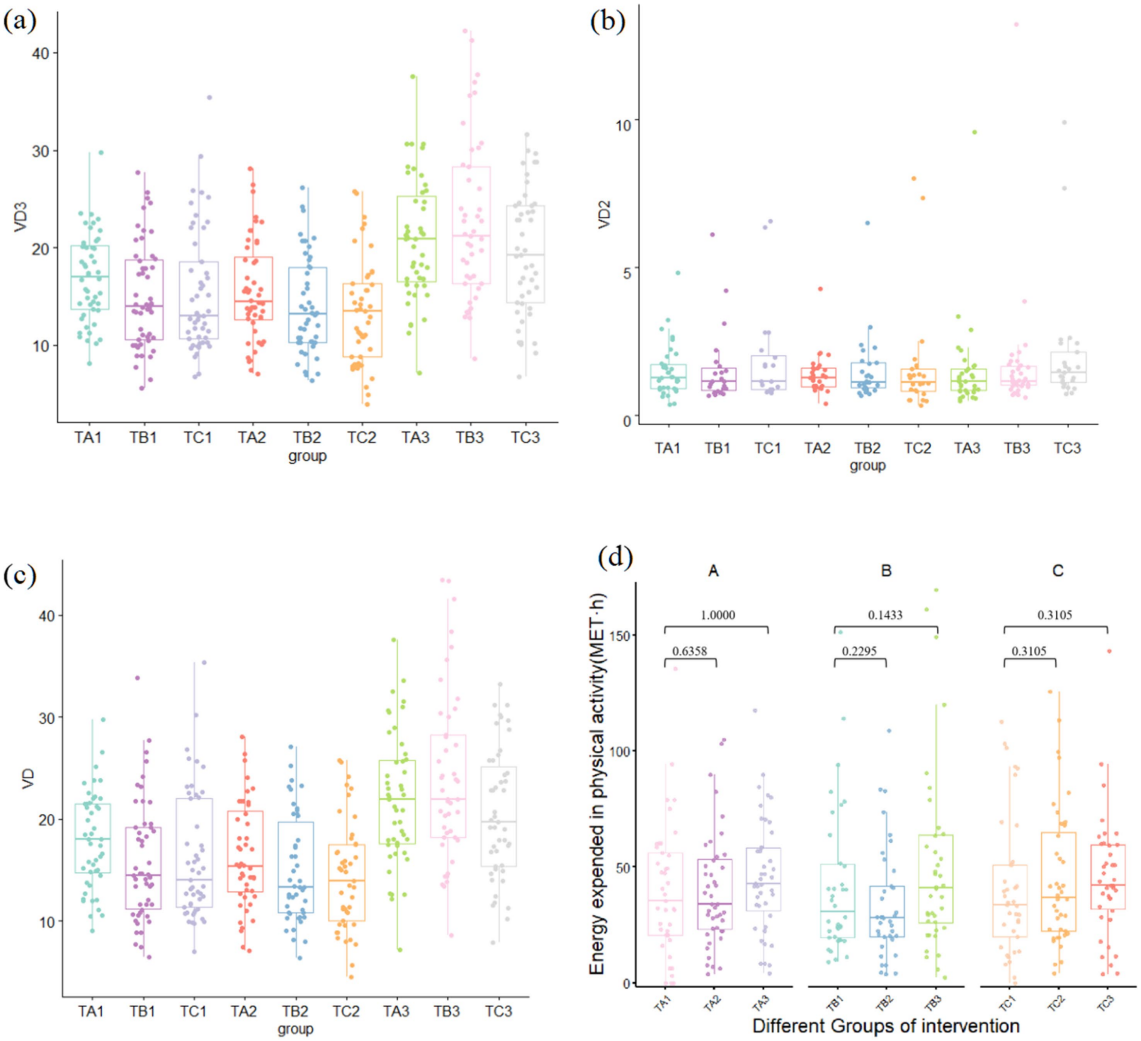
Vitamin D levels and physical activity levels in study groups across different time periods: **(a)** 25-OH VD3 level, **(b)** serum 25-OH VD2, **(c)** total 25-OH VD, and **(d)** physical activity level.

Analysis of serum vitamin D levels revealed the following distinct temporal patterns:

- Short-term (6 weeks): No significant changes in 25-OH VD_3_ or total 25-OH VD levels were observed in any group compared with baseline, indicating stability during the initial intervention phase.
- Long-term (12 weeks): By the end of the intervention, compared with baseline, both intervention groups exhibited significant increases in 25-OH VD_3_ and 25-OH VD concentrations, with Group B (100 g/day) exhibiting a more pronounced increase than Group A (50 g/day). In contrast, the increase in the control group was modest and remained near the lower threshold of the normal range (20 ng/mL) (Figures 1a–c).

Furthermore, because this field study was conducted in Beijing between June and September, a matched analysis of the physical activity of the participants in each group was conducted. The results showed no significant differences in activity levels at baseline, 6 weeks, or 12 weeks across any groups during the intervention period (*p* > 0.05) (Figure 1d).

### 3.2 Comparison of vitamin D levels in participants within the same group at different time periods

#### 3.2.1 Percentage of participants with normal levels of vitamin D

The distribution of participants with normal serum 25-OH VD levels in the intervention and control groups at different time points is shown in Table 4. In both intervention groups (A and B), the number of participants with normal 25-OH VD levels increased progressively from baseline to the interim assessment and further to the final assessment. These increases were statistically significant (*p* < 0.0001). In control group C, the number of participants with normal levels of 25-OH VD in the final assessment was significantly higher than that in the interim and baseline assessments (*p* < 0.0078). At baseline, the normalisation rate in the control group did not differ from that in the two intervention groups. However, after 12 weeks of whole-grain dietary intervention, the normalisation rates in both intervention groups were higher than those in the control group.

**Table 4:** Distribution of participants with normal levels of 25-OH VD in each group at different time periods.

Group*	Number	Normalisation rate (%)	*χ*^2^	*p*
A1	48	16.7	19.07	<0.0001
A2	48	27.1		
A3	47	57.4		
B1	50	20.0	25.57	<0.0001
B2	46	26.1		
B3	45	66.7		
C1	49	20.5	9.70	0.0078
C2	45	17.8		
C3	47	46.8		

* 1, 2, and 3 represent baseline, 6 weeks of intervention, and 12 weeks of intervention, respectively.

#### 3.2.2 Comparison within group

**Figure 2:**
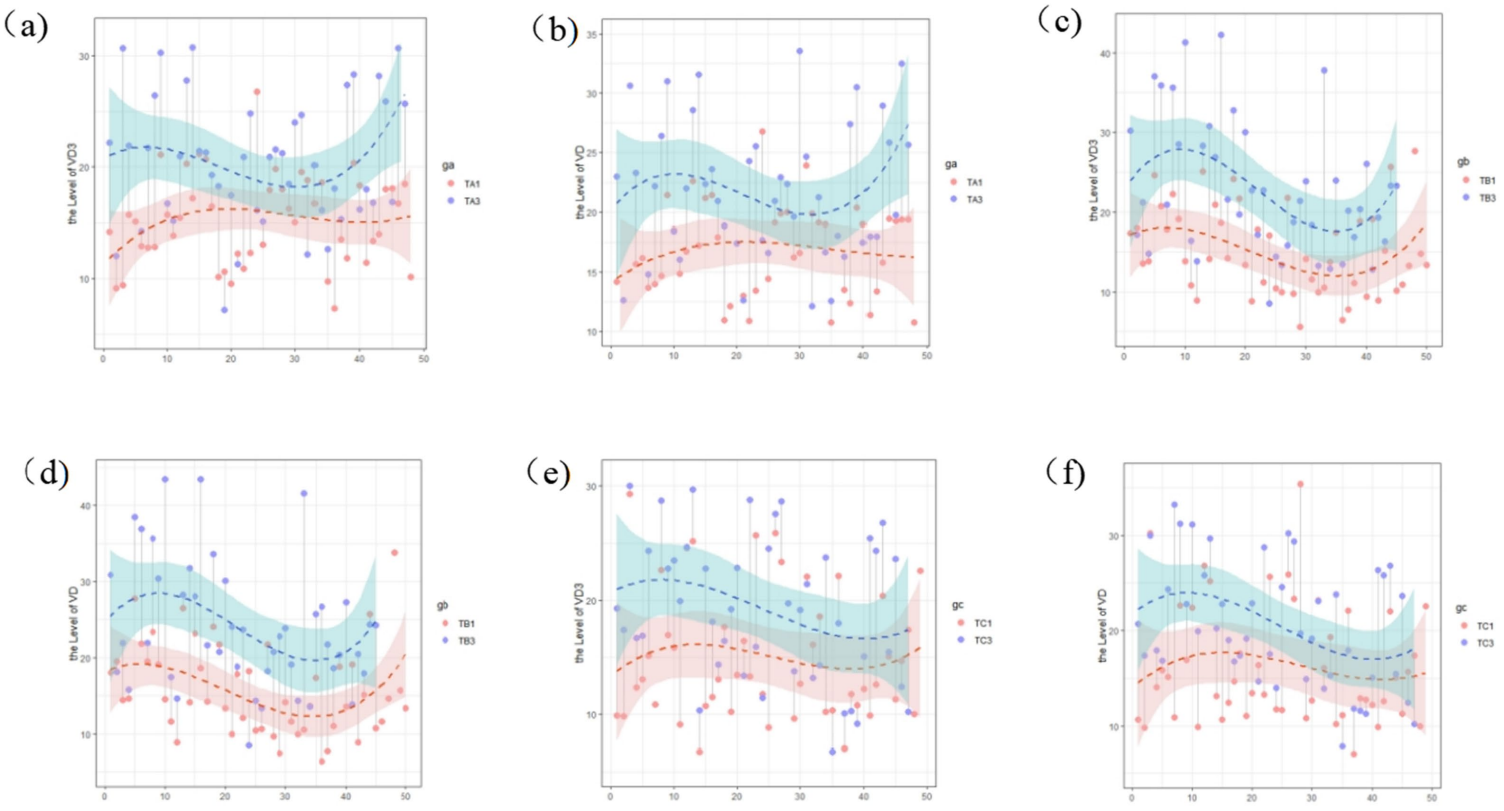
Changes in the vitamin D level within study groups across different time periods: **(a)** 25-OH VD_3_ at baseline and final assay in intervention group A; **(b)** total 25-OH VD at baseline and final assay for participants in intervention group A; **(c)** 25-OH VD_3_ at baseline, final assay for participants in intervention group B; **(d)** total 25-OH VD at baseline, final assay for participants in intervention group B; **(e)** 25-OH VD_3_ at baseline, final assay for participants in control group C; and **(f)** 25-OH VD at baseline, final assay for participants in control group C.

Within-group comparisons of serum 25-OH VD_3_ and 25-OH VD levels showed broadly consistent trends across the intervention and control groups, with vitamin D levels being significantly higher than those in the baseline data (Figure 2). There were no significant differences between the baseline and interim assay results for each group (*p* > 0.05). The 25-OH VD_3_ and 25-OH VD levels were significantly higher in all groups in the final assay, and the differences between the baseline and interim data were statistically significant (*p* < 0.05) (Table 5).

**Table 5:** Comparison of vitamin D levels among participants within groups at different time periods.

Group*	VD3		VD	
	Wilcoxon Z	*p*	Wilcoxon Z	*p*
A1 vs. A2	−0.1685	0.9845	−0.1685	0.9845
A1 vs. A3	−4.6002	<0.0001	−4.4141	<0.0001
A2 vs. A3	−4.0940	<0.0001	−4.2057	<0.0001
B1 vs. B2	0.9021	0.6390	0.7481	0.7348
B1 vs. B3	−4.5094	<0.0001	−4.9044	<0.0001
B2 vs. B3	−5.1994	<0.0001	−5.4375	<0.0001
C1 vs. C2	1.2602	0.4178	1.1996	0.4534
C1 vs. C3	−3.1846	0.0041	−3.4704	0.0015
C2 vs. C3	−4.2139	<0.0001	−4.4092	<0.0001

1, 2, and 3 represent baseline, 6 weeks of intervention, and 12 weeks of intervention, respectively. * A, B, and C denote intervention group A, intervention group B, and control group, respectively.

### 3.3 Comparison amongst different groups in the same period

Serum 25-OH VD_3_, 25-OH VD_2_, and 25-OH VD levels were compared across groups at each intervention period, with no significant between-group differences in 25-OH VD_2_ levels observed at any time point. Based on the baseline and interim data, the results of the two-by-two comparisons between different groups of the three vitamin D indicators showed no statistically significant differences amongst the groups (*p* > 0.05). However, in the final data, serum 25-OH VD_3_ levels in the intervention group B were significantly higher than those in the control group C, as were the results of the between-group comparisons of 25-OH VD (25-OH VD_3_, *p* = 0.0357, 25-OH VD, *p* = 0.0256). No significant differences were noted between intervention groups A and B and between intervention group A and control group C (Table 6).

**Table 6:** Comparison among different groups in the same period.

Group	VD3		VD2		VD	
	ANOVA F	*p*	Wilcoxon Z	*p*	ANOVA F	*p*
A1 vs. C1	0.03	0.8675	0.6925	0.4886	0.61	0.4373
B1 vs. C1	0.07	0.7930	0.8157	0.4147	0.09	0.7650
A1 vs. B1	0.02	0.8932	0.1545	0.8772	1.74	0.1916
A2 vs. C2	3.75	0.0558	−0.772	0.4401	3.56	0.0623
B2 vs. C2	0.36	0.5479	−0.5129	0.6080	0.33	0.5672
A2 vs. B2	1.91	0.1698	−0.1968	0.8440	0.11	0.7442
A3 vs. C3	1.22	0.2728	1.892	0.0585	1.12	0.2918
B3 vs. C3	4.55	0.0357	1.5911	0.1116	5.15	0.0256
A3 vs. B3	1.49	0.2261	−0.7328	0.4637	1.86	0.1764

1, 2, and 3 represent baseline, 6 weeks of intervention, and 12 weeks of intervention, respectively. * A, B, and C denote intervention group A, intervention group B, and control group, respectively.

## 4 Discussion

Whole grains, as alternatives to refined grains, include intact kernels or processed forms, such as milled, crushed, or flaked products. Whole grains retain all parts of the grain kernel, such as the endosperm, germ, and bran, and are rich in dietary fibre, B vitamins, minerals, and phytoactives, offering high nutritional value and bioactive properties. Evidence from domestic and foreign research suggests that regular consumption of whole grains reduces the risk of diabetes, cardiovascular disease, cancer, and other chronic conditions. It also helps maintain a healthy body weight and helps lower the level of inflammation in the body (11). However, in 2015, the average whole-grain intake in Chinese urban and rural residents was 20.3 g per standard person day (12). China’s nutritional monitoring data indicate an average whole-grain intake amongst Chinese adults of 20.1 g/day, which is significantly lower than the standard 50–150 g consumption of whole grains and legumes per day, as recommended by the Dietary Guidelines for Chinese Residents, 2022 (13). Additionally, the Global Burden of Disease report showed that, in China, insufficient intake of whole grains is ranked second amongst the causes of high mortality from diseases caused by suboptimal dietary patterns (14). With improving living standards and an ageing population, the prevalence of diabetes amongst Chinese adults is rapidly increasing, accompanied by an increase in diabetes-related mortality (15, 16). Epidemiological studies have shown that increased intake of whole grains can reduce total and disease-specific mortalities (17).

Vitamin D plays a crucial role in blood glucose regulation. Vitamin D deficiency may impair insulin sensitivity and induce insulin resistance, thereby increasing the risk of diabetes (18). Appropriate vitamin D supplementation can enhance the body’s sensitivity to insulin, improve glucose metabolism, and facilitate the uptake and utilisation of glucose in the blood, which helps maintain normal blood glucose levels and reduces the risk of the progression of pre-diabetic symptoms to type 2 diabetes (19). Vitamin D has a protective effect on pancreatic *β*-cells—it reduces apoptosis of pancreatic islet cells, improves islet cell graft survival, and promotes insulin secretion (20). Additionally, vitamin D metabolites in the body regulate blood glucose concentration, which can effectively prevent hyperglycaemia. Vitamin D also has anti-inflammatory and antioxidant effects, which help reduce the damage caused by oxidative stress and autoimmunity to pancreatic islet β-cells and even to organs throughout the body, thus reducing the risk of diabetic complications, such as cardiovascular disease, retinopathy, and peripheral neuropathy (21).

Based on the above discussion, this study conducted a whole-grain intervention programme to evaluate changes in serum vitamin D levels amongst pre-diabetic adults in China. Serum 25-OH VD concentrations were determined via this protocol: samples were purified and concentrated after pretreatment, separated by liquid chromatography, detected by tandem MS in multiple reaction monitoring mode, and quantified using the isotope internal standard method. One quality control product was added to every 20 samples during the detection process to ensure the quality of the entire batch. This method can accurately determine the concentrations of 25-OH VD_3_ and 25-OH VD_2_ in the samples.

At baseline and interim assessments, participants’ serum vitamin D levels averaged approximately 15 ng/mL, indicating insufficiency. However, after 12 weeks of intervention, an increasing trend in vitamin D concentration was observed in both the intervention and control groups, and the mean values in the two intervention groups exceeded 20 ng/mL, reaching the threshold for adequacy.

A comparison of serum 25-OH VD_3_ and 25-OH VD concentrations across groups and time points showed that serum 25-OH VD concentrations remained relatively stable after 6 weeks of whole-grain dietary intervention, with no statistically significant difference from baseline. Vitamin D levels did not change significantly in the participants during the whole-grain dietary intervention, likely due to the fact that the low-fat percentage of whole-grain staples replaced some of the refined-grain staples. Whole-grain dietary patterns did not affect the absorption or utilisation of the fat-soluble vitamin D in pre-diabetic populations. Compared with baseline, at the 6-week stage of the intervention, the levels of 25-OH VD concentration in all groups of this study were relatively stable, but the mean values indicated an insufficient level. Final monitoring after 12 weeks of intervention showed a statistically significant increase in serum 25-OH VD concentrations in participants in both intervention groups compared with baseline and mid-term values. Based on the final monitoring data of different groups, 25-OH VD levels in participants from both intervention groups exceeded those in the control group. The mean values of both intervention groups reached the reference threshold of adequacy, whereas the mean value of the control group remained at the borderline of the reference threshold of adequacy. These results indicate that adherence to the long-term intake of whole grains positively influences nutrient absorption and the metabolic milieu of 25-OH VD in the organism.

Whole grains are rich in dietary fibre, minerals, and other nutrients that contribute to a favourable intestinal environment, aiding the absorption of fat-soluble vitamins. Whole-grain dietary intervention promotes the enrichment of specific beneficial gut microbiota, which ferment dietary fibre into small-molecule metabolites with important physiological activities, such as short-chain fatty acids (SCFAs), and also releases free polyphenols (22). Both dietary fibre and polyphenols modulate gut health: fibre effectively increases the abundance of beneficial bacteria, whilst polyphenols regulate gut microbiota primarily by inhibiting pathogenic bacteria (e.g., *Staphylococcus aureus* and *Salmonella typhimurium*) and promoting beneficial bacteria (23). An interaction also exists between vitamin D and gut microbiota: vitamin D exerts a role in regulating gut microbiota composition by inhibiting the growth of pathogenic bacteria whilst nourishing beneficial bacterial strains (24). Furthermore, their effects on the gut are also reflected in improving intestinal barrier properties and alleviating systemic inflammatory status—specifically, reducing intestinal barrier dysfunction induced by inflammation and oxidative stress, enhancing barrier integrity, and strengthening the intestinal absorption and metabolic capacity of nutrients including vitamin D. Gut microbiota regulate endocrine vitamin D metabolism via fibroblast growth factor 23, ultimately restoring homeostasis; this restoration includes increasing the levels of 25-hydroxyvitamin D, 24,25-dihydroxyvitamin D, and 1,25-dihydroxyvitamin D (25). A previous study found that the gut microbe *Carnobacterium maltaromaticum* (*C. maltaromaticum*) and its metabolites may be involved in the metabolism and utilisation of vitamin D. It colonises the gut in an oestrogen-dependent manner and, together with other microbes, enhances intestinal vitamin D production (26). These beneficial bacteria can further regulate vitamin D metabolism and activate and strengthen vitamin D receptors to maintain host health (27).

Whole grains are rich in minerals such as magnesium (Mg) and zinc (Zn). As absorption cofactors, Mg and Zn provided by whole grains are crucial for the normal metabolism and function of vitamin D. Regular intake of whole grains enhances higher mineral absorption and bioavailability (28). The activation of vitamin D requires hydroxylation reactions in the liver and kidneys, and the activity of enzymes involved in these reactions is Mg-dependent. Thus, inadequate Mg levels in the body can prevent vitamin D from being converted into its biologically active form. Furthermore, Zn may exert a synergistic effect on vitamin D and participate in the metabolism and functional exertion of vitamin D (29).

Meanwhile, the final measurements also revealed a statistically significant increase in serum 25-OH VD concentrations amongst participants in the control group, which may be attributed to complex environmental factors, particularly sunlight exposure. The duration of sunlight and intensity of ultraviolet (UV) radiation differ across various latitudes and longitudes; even at the same latitude and longitude, both the duration of sunlight and the intensity of UV radiation vary by month. Ultraviolet radiation is essential for cutaneous vitamin D synthesis, as it mediates the conversion of 7-dehydrocholesterol in the skin to vitamin D. Experimental studies investigating the effects of simulated solar ultraviolet radiation (UVR) on circulating 25-OH VD3 levels in young and older individuals have demonstrated that UVR exposure significantly elevates serum 25-OH VD3 concentrations (30, 31). In China, vitamin D deficiency and insufficiency are highly prevalent during spring and winter. However, this study was conducted in Beijing between June and September, which coincided with summer and early autumn, wherein the region experiences extended daylight hours (approximately 14–15 h in June) and high ultraviolet radiation intensity. Serum 25-hydroxyvitamin D levels are determined by multiple factors, including UV intensity and the opportunity for direct sunlight exposure. Outdoor physical activity during this season can further enhance vitamin D levels (32). Due to the intense summer heat in June and July, opportunities for outdoor activity were relatively limited. However, serum 25-OH VD levels in the general population typically peak in September, which coincides with the most climatically pleasant months in Beijing (September–October). Favourable weather conditions during this period promote increased outdoor activity, thereby enhancing endogenous vitamin D synthesis (33). This observation is consistent with the changes in 25-OH VD levels observed in the control group throughout the baseline, mid-term, and final monitoring timepoints. To account for these potential confounding factors, participants’ physical activity data from baseline to the final stage were analysed, specifically focusing on the 1 week preceding each survey date. The results indicated no significant differences in the physical activity levels of the participants amongst baseline, 6 weeks of intervention, and 12 weeks of intervention across all groups. This finding suggests that, although seasonal sunlight exposure may have contributed to the overall increase in serum vitamin D levels in all groups, it did not bias the intergroup comparisons. The significant differences in vitamin D levels between the intervention and control groups at 12 weeks likely reflected the specific effects of whole-grain intake, rather than external factors.

This study has some limitations. First, due to constraints in product manufacturing technology, the whole-grain foods used differed significantly from non-whole-grain foods in terms of appearance, colour, and taste, making it impossible to implement a blinded intervention. Future studies should strengthen the communication with whole-grain food manufacturers to optimise processing technologies and conduct double-blind controlled intervention trials. Second, considering cost and feasibility, the intervention duration was limited to 12 weeks to minimise the burden on participants and ensure adherence. Longer-term studies with larger sample sizes are needed to validate these findings and evaluate their sustained effects. Third, efforts should be made to collect and analyse data such as dietary vitamin D intake and participants’ sunlight exposure, maintain detailed records, and ensure in-process quality control. These measures will help validate the current findings and explore the long-term effects of whole-grain intake on vitamin D metabolism.

## 5 Conclusion

This study provides valuable insights into the relationship between whole-grain interventions and serum vitamin D levels amongst individuals at risk of diabetes. This randomised controlled trial demonstrated that increased intake of whole grains can significantly improve vitamin D status in pre-diabetic populations. These findings highlight the potential of dietary modifications for diabetes prevention and management and provide a basis for the development of public health policies and clinical guidelines.

In summary, whole-grain consumption may be a promising strategy for improving vitamin D status and reducing the risk of diabetes. Further studies are required to confirm these findings and explore the underlying mechanisms. More research should be conducted to test various indicators of internal homeostasis of body metabolism to further explore the effects of a whole-grain diet on health and on risk factors related to metabolic syndrome. Such evidence will provide a stronger scientific foundation for improving the health of people at risk of diabetes mellitus and related metabolic syndromes.

## Data Availability

The raw data supporting the conclusions of this article will be made available by the authors, without undue reservation.
